# Are There Hidden Genes in DNA/RNA Vaccines?

**DOI:** 10.3389/fimmu.2022.801915

**Published:** 2022-02-08

**Authors:** Christopher A. Beaudoin, Martin Bartas, Adriana Volná, Petr Pečinka, Tom L. Blundell

**Affiliations:** ^1^ Department of Biochemistry, Sanger Building, University of Cambridge, Cambridge, United Kingdom; ^2^ Department of Biology and Ecology, University of Ostrava, Ostrava, Czechia; ^3^ Department of Physics, University of Ostrava, Ostrava, Czechia

**Keywords:** SARS-CoV-2, spike protein, DNA vaccine, RNA vaccine, ORF2b, ORF-Sh, codon optimization

## Abstract

Due to the fast global spreading of the Severe Acute Respiratory Syndrome Coronavirus – 2 (SARS-CoV-2), prevention and treatment options are direly needed in order to control infection-related morbidity, mortality, and economic losses. Although drug and inactivated and attenuated virus vaccine development can require significant amounts of time and resources, DNA and RNA vaccines offer a quick, simple, and cheap treatment alternative, even when produced on a large scale. The spike protein, which has been shown as the most antigenic SARS-CoV-2 protein, has been widely selected as the target of choice for DNA/RNA vaccines. Vaccination campaigns have reported high vaccination rates and protection, but numerous unintended effects, ranging from muscle pain to death, have led to concerns about the safety of RNA/DNA vaccines. In parallel to these studies, several open reading frames (ORFs) have been found to be overlapping SARS-CoV-2 accessory genes, two of which, ORF2b and ORF-Sh, overlap the spike protein sequence. Thus, the presence of these, and potentially other ORFs on SARS-CoV-2 DNA/RNA vaccines, could lead to the translation of undesired proteins during vaccination. Herein, we discuss the translation of overlapping genes in connection with DNA/RNA vaccines. Two mRNA vaccine spike protein sequences, which have been made publicly-available, were compared to the wild-type sequence in order to uncover possible differences in putative overlapping ORFs. Notably, the Moderna mRNA-1273 vaccine sequence is predicted to contain no frameshifted ORFs on the positive sense strand, which highlights the utility of codon optimization in DNA/RNA vaccine design to remove undesired overlapping ORFs. Since little information is available on ORF2b or ORF-Sh, we use structural bioinformatics techniques to investigate the structure-function relationship of these proteins. The presence of putative ORFs on DNA/RNA vaccine candidates implies that overlapping genes may contribute to the translation of smaller peptides, potentially leading to unintended clinical outcomes, and that the protein-coding potential of DNA/RNA vaccines should be rigorously examined prior to administration.

## Introduction

The Severe Acute Respiratory Syndrome Coronavirus 2 (SARS-CoV-2) is a positive-sense single-stranded RNA virus that was first described in late 2019 (1). SARS-CoV-2 is phylogenetically related to the causative agent of the 2002 SARS-CoV epidemic and causes many of the same symptoms, such as fever and myalgia (2). Because of the high transmissibility of SARS-CoV-2 and rapid spreading throughout the world, by March 2020, the World Health Organization declared the global outbreak as the COVID-19 pandemic (3). The health and economic-related losses accruing as a result of the pandemic led to the prioritization of prevention and treatment options with the quickest route to safe clinical application (4). Although small molecule inhibitors and inactivated or live attenuated virus vaccine candidates have been used to successfully treat infection by pathogenic viruses, the pipelines to bring these products into clinical use can require significant time and resources with potentially low success rates (5, 6). However, among novel vaccine delivery platforms developed in recent years, DNA and RNA vaccines have become of interest due to their potential to be inexpensively and quickly produced at a large scale (7). Only the nucleotide sequence of the selected antigenic protein is required to begin production, which can be derived from DNA/RNA sequencing of the virus. Thus, DNA/RNA vaccines have been suggested as prime candidates for mitigating COVID-19 transmission.

The SARS-CoV-2 genome codes for at least 30 proteins, three of which are exposed on the virion surface and can be recognized by the immune cell system (8–10). The spike protein is a large trimeric glycoprotein (1,273 amino acid long protomers) that protrudes from the virion surface to bind to cell surface receptors on host cells, such as angiotensin converting enzyme II (ACE2), in order to initiate viral entry (11). The large surface area of the spike protein and its role in host cell entry make it attractive as a target for the immune system and clinical treatments, such as drugs and therapeutic antibodies (12). Of note, the spike protein is heavily glycosylated, which helps shield the virus from interactions with antibodies (13). The region with the lowest degree of glycosylation is the receptor-binding domain, which binds to host cell surface proteins to initiate viral entry, and, as a result, is the most antigenic region of the spike protein (14). The other two proteins exposed on the virion surface, the envelope and membrane proteins, are also available for use as antigen targets; however, they are smaller in size and less accessible for protein-protein interactions than the spike protein. Because of the evidence indicating that spike is the most suitable antigenic target for SARS-CoV-2, it has been widely used in vaccine trials.

Numerous companies and academic institutions across the globe have developed or are currently developing DNA/RNA vaccines for the SARS-CoV-2 spike protein (15). Generally, in the case of DNA vaccines, the full-length SARS-CoV-2 spike protein DNA sequence is inserted into a plasmid, and additional technologies, such as electroporation, can assist in making transfection more efficient (16, 17). The spike protein DNA transfected into the human cell can then be transcribed and translated to create the trimeric spike protein, which, then, moves to the endoplasmic reticulum and Golgi apparatus for post-translational modification (e.g. signal sequence cleavage, glycosylation) and continues through the secretory route to become anchored to the cell membrane for exposure to the immune system (18, 19)⁠. The RNA-based vaccine formulations comprise lipid nanoparticles assembled around mRNA molecules coding for the full-length SARS-CoV-2 spike sequence (20). The transfected mRNA can be directly translated to make the spike protein. The BioNTech/Pfizer and Moderna mRNA vaccines, which have widely been approved by government agencies and administered in several countries, have reported approximately 50-70 and 70-90% effectiveness after 1 and 2 doses, respectively, against the wild-type and alpha variant (B.1.1.7) and 30-60 and 60-90% effectiveness, respectively, against the beta (B.1.351) and gamma (P.1) variants (21, 22). However, additional variants of concern have been noted to provide either further partial or complete immune escape; thus, adapting the sequences may be required over time (23, 24). Prior to COVID-19, no DNA/RNA vaccines had been approved for human use, but, in August 2021, BioNTech and Pfizer received FDA approval for use of their mRNA vaccine (25). Further investigation into nucleic acid-based vaccine delivery platforms may improve effectiveness.

Although SARS-CoV-2 DNA/RNA vaccines have been subjected to health and safety testing prior to bulk dissemination, a diverse assortment of both systemic and local (near injection site) side effects, ranging from mild to severe, following vaccination have been described (26, 27). Symptoms resembling that of viral infection (e.g. headache and myalgia), life-threatening conditions (e.g. myocardial injury and thrombosis), and mortalities have been reported in relation to vaccination (28–31). Although some side effects may stem from the delivery modalities, several studies have indicated that the spike protein alone causes adverse effects on host tissues, such as blood brain barrier disruption, neuron fusion, inflammation, and cell senescence (32–35). Although it is difficult to detect the origin of side effects in vaccinated individuals, more investigation on the cellular effects of mRNA vaccines or the expressed protein antigen are warranted to create safer vaccines.

## Overlapping ORFs on the SARS-CoV-2 Spike Protein Nucleotide Sequence

The understanding that one mRNA codes for one gene in eukaryotes, not considering alternative splicing, has been challenged after several studies have revealed the presence and translation of multiple open reading frames (ORFs) within one expressed mRNA (36, 37). Additionally, stretches of RNA that have been annotated as non-coding have also been discovered to code for small peptides from internal ORFs in the larger gene that have regulatory activity in the cell (38, 39). Alternative start codons, internal ribosome entry sites, and frameshifting have all been described as mechanisms contributing to the translation of smaller ORFs within a larger expressed gene (40–42). Furthermore, overlapping genes are a common feature among viral genomes (43). Overlapping genes in viruses originate from mutations that allow the spontaneous generation of a translation start site within a gene that leads often to a new ORF usually with a different frame while still maintaining the integrity of the original gene (44). Thus, understanding the coding capacity of a viral transcript in a human cell may be more complex than assessing the full-length protein sequence alone.

Several ORFs have been found to overlap previously annotated genes in the SARS-CoV-2 proteome (45). For instance, ORF3d and ORF9c have been recently discovered, using Ribo-Seq and phylogenetics analyses, to overlap the ORF3a and N genes, respectively (8, 9). Included among the newly discovered overlapping ORFs in the SARS-CoV-2 genome are ORF2b and ORF-Sh, which have been shown to overlap the spike protein sequence on the +1 frame (one nucleotide towards the 3’ end) (9, 46, 47). The ORF2b and ORF-Sh nucleotide sequences are both 120 nucleotides long and both code for 39 amino acid long proteins. ORF2b has been found to be translated in human cells during infection, and ORF-Sh has thus far been found only using in-depth phylogenetic comparisons. ORF2b was found to be absent in almost all bat coronavirus strains in a genomic comparison study, suggesting that it has recently evolved (48). ORF-Sh has been proposed to have evolved recently among the clade of viruses that includes the Bat-CoV-RaTG13 and pangolin coronavirus strains. Mutations in only few sequenced SARS-CoV-2 strains have been discovered that lead to truncation of the protein sequence (47, 49). Little is known about the structure or function of these proteins, although both are predicted to contain a transmembrane domain. The existence of additional open reading frames within the sequence of the spike protein sequence, however, begs the question as to whether these or other overlapping ORFs are being translated on DNA/RNA vaccine sequences. Since the translation of overlapping accessory ORFs has been shown to significantly alter the dynamics of host protein-protein interaction networks, the translation of these two, and possibly other ORFs within the sequence of the spike protein RNA/DNA vaccines, may lead to signaling perturbations that resemble SARS-CoV-2 infection (50, 51).

**Figure 1:**
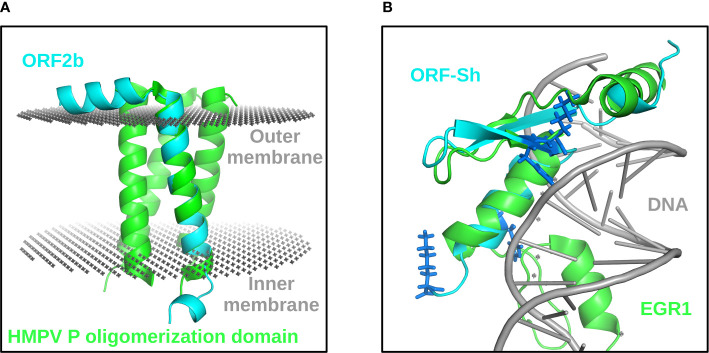
Structural characterization of ORF2b and ORF-Sh. Full-length ORF2b (cyan) aligned with one protomer of the HMPV P oligomerization domain (green) fixed in a bilayer membrane (gray) is depicted **(A)**. ORF-Sh (cyan) aligned to the mouse early growth response protein 1 (EGR1) (PDB: 1p47) (green), which is a homolog of transcriptional repressor CTCF, bound to DNA (grey) is shown **(B)**. Basic residues in the ORF-Sh protein are shown as dark blue sticks **(B)**.

Since little has been reported on the functionality of ORF2b or ORF-Sh in infected cells (although translation of ORF-Sh is still to be confirmed) and no homologous domains were found using sequence analysis tools, we used structural bioinformatics methodologies, as previously performed to elucidate the three-dimensional features of the SARS-CoV-2 proteome, to model the structural characteristics both ORFs (52–61). As shown in **Figure 1A**, the majority of the ORF2b protein is predicted to be fixed in the membrane (62)⁠. Structural similarity comparisons revealed that the predicted ORF2b transmembrane domain resembles the human metapneumovirus phosphoprotein (HMPV) P oligomerization domain (PDB: 5oix; TM-score: 0.67), which may result in tetramerization or other states of oligomerization (**Figure 1A**) (63). Such oligomerization in the membrane could lead to viroporin activity – similar to the ORF3a and E SARS-CoV-2 proteins (64, 65). Although a transmembrane domain was predicted in the ORF-Sh sequence, structural modelling and secondary structure prediction depict a bend in the middle of the putative transmembrane domain (**Figure 1B**). Structural comparisons revealed fold similarity between the predicted ORF-Sh model and DNA-binding zinc finger proteins, such as the transcriptional repressor CTCF (PDB: 1x6h; TM-score: 0.38), which is further supported by the presence of four basic amino acids on what is predicted to be the DNA-exposed side shown in **Figure 1B** (66). Experimental validation is required to verify these results however. Nevertheless, the translation of overlapping, small ORFs within larger ORFs can result in harmful effects on host tissues, such as interfering with organelle membrane protein activity or perturbing signaling pathways. Thus, the protein-coding potential of DNA/RNA vaccines within the context of overlapping ORFs should be investigated further.

## Codon Optimization of DNA/RNA Vaccine Candidates

Although the presence of overlapping genes on the wild-type nucleotide sequence of the spike protein challenges the effectiveness of DNA/RNA vaccines, precautionary steps can be taken to prevent the translation of these smaller, internal ORFs. For example, vaccine nucleotide sequences can be selectively codon optimized, as is normally performed to enhance translation efficiency in host tissues, to remove alternative start codons and internal ribosome entry sites, thus preventing non-specific recognition by ribosomal complexes (67). Codon optimization without consideration of overlapping ORFs, however, can result in both disruption of the current overlapping ORFs, ORF2b and ORF-Sh in the case of the spike protein vaccines, or spontaneously generating new ORFs. Although most, if not all, DNA/RNA vaccine candidate spike sequences have been reported to be codon optimized for translation in human cells, the spike nucleotide sequences have largely, so far, been kept private by the corresponding company or institution. Interestingly, however, the Moderna mRNA-1273 and Pfizer BNT162b2 vaccine mRNA sequences have been made publicly-available (https://github.com/NAalytics; https://berthub.eu/articles/posts/reverse-engineering-source-code-of-the-biontech-pfizer-vaccine/). The posting of these data allows direct comparative analyses between the vaccine-formulated and wild-type spike protein sequences.

Comparing the nucleotide sequences of the wild-type and vaccine mRNA spike proteins may reveal the extent to which the sequences have been changed during codon optimization, thus potentially altering translation efficiency of the spike protein and overlapping ORFs. Of note, prior to codon optimization, both companies have reported including proline mutations to stabilize and preserve the spike protein structure, thus implying small changes in spike amino acid content as well. Using the EMBOSS Needle pairwise sequence alignment tool, the wild-type spike sequence (NCBI accession: NC_045512) is found to be 68.7% and 45.3% identical to the mRNA-1273 and BNT162b2 vaccine spike sequences (as opposed to the entirety of the mRNA sequence), respectively, and the mRNA-1273 and BNT162b2 spike sequences are 48.6% identical to one another (68). The GC contents of the wild-type, BNT162b2, and mRNA-1273 spike nucleotide sequence, which correlate well with translation efficiency, are 37.3%, 56.9%, and 62.3%, respectively. These alignments reveal that extensive codon optimization was performed during vaccine preparation.

To quantify the degree to which the codon optimization performed on the vaccine mRNA sequences matches that of the human genome amino acid pool, the codon adaptability index (CAI), which has been noted to be an accurate reflector of gene translation, was calculated for all three spike sequences using the COUSIN and CAIcal web servers (69–71). As a reference, calculated CAI values for SARS-CoV-2 genes with regards to human codon usage average around 0.7, and a higher score represents a stronger indication for translation (72, 73). The CAI values for the wild-type, BNT162b2, and mRNA-1273 spike nucleotide sequences are 0.703, 0.715, and 0.981, respectively. While the BNT162b2 vaccine CAI value was slightly increased compared to the wild-type sequence, the mRNA-1273 vaccine CAI value was found to be significantly higher – almost reaching the maximum value. These findings suggest that the codon optimization used on both vaccine sequences have resulted in higher translation potential than the wild-type. Notably, the mRNA-1273 vaccine codon usage seems much more closely aligned with human codon biases, and the sequence contains a lower amount of substituted nucleotides and a higher GC content.

## Overlapping ORFs on DNA/RNA Vaccine Candidates

Considering the extensive codon optimization performed on the vaccine spike sequences, the comparison of putative ORFs in the wild-type and selected vaccine mRNA sequences may shed light on the protein-coding potential of DNA/RNA sequences used in SARS-CoV-2 vaccines. Thus, in order to examine the differences between the available ORFs on the wild-type, Moderna mRNA-1273, and Pfizer BNT162b2 sequences, putative ORFs of all three nucleotide sequences were detected using the NCBI ORFfinder web server (https://www.ncbi.nlm.nih.gov/orffinder/). Although ORF identification using this tool does not imply translation, an overview of the available reading frames may provide insights into coding potential differences between the wild-type and vaccine candidate sequences. Minimum ORF length was set to the default 75 nucleotides, no alternative initiation codons were allowed, and only “ATG” start sites were considered.

**Figure 2:**
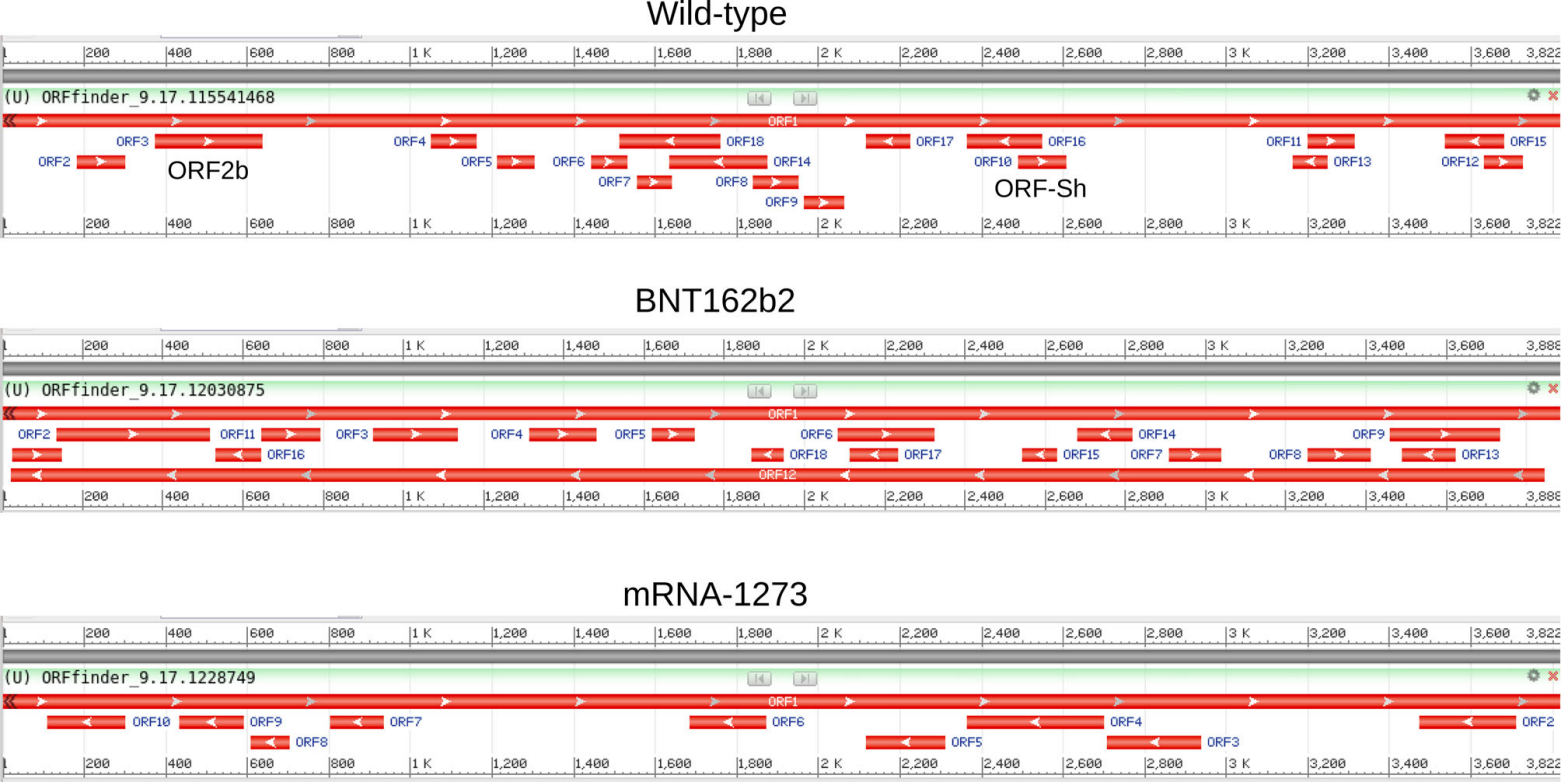
ORFs detected on the wild-type, BNT162b2, and mRNA-1273 spike sequences. The ORFs identified using NCBI ORFfinder for the ORFs detected on the wild-type (top), BNT162b2 (middle), and mRNA-1273 (top) spike sequences are depicted. The presence of ORF2b and ORF-Sh (ORF3 and ORF10, respectively, in the NCBI ORFfinder output) are noted on the wild-type sequence (top).

As shown in **Figure 2**, ORF2b and ORF-Sh were found in the wild-type sequence; however, both ORFs are absent in both of the mRNA vaccine candidate sequences. The counts, lengths, and sequence identities of predicted ORFs on both mRNA sequences were found to be markedly different from one another and from the wild-type, re-asserting that codon optimization can result in significant changes in the presence of overlapping ORFs. Eleven small overlapping ORFs (27-87 residues long) were discovered using NCBI ORFfinder on the wild-type spike protein sequence, and eight small ORFs (26-52 residues long) were found to overlap the Pfizer BNT162b2 vaccine mRNA sequence. Notably, the Moderna mRNA-1273 vaccine mRNA sequence displayed no overlapping sequences on the positive sense strand – only on the negative sense, which can be disregarded when considering mRNA. However, DNA-based vaccines, such the INO-4800 SARS-CoV-2 spike DNA vaccine from INOVIO Pharmaceuticals, should be assessed for the presence of protein-coding ORFs on the reverse strand (73). Thus, in terms of predicted protein-coding potential, the Moderna mRNA-1273 mRNA vaccine appears to be the most optimized sequence of the two to solely code for the SARS-CoV-2 spike protein. These findings also support the notion that vaccine candidate sequence codons can be reliably edited to remove undesired ORFs. Newly predicted ORFs on the Pfizer BNT162b2 vaccine mRNA sequence, on the other hand, highlight the fact that codon optimization can also lead to the spontaneous generation of novel overlapping ORFs. Of interest is the observation that allowing detection of alternative initiation codons increased the number of predicted ORFs on the positive sense strand of the wild-type (11 to 19 ORFs), BNT162b2 (8 to 25), and mRNA-1273 (0 to 4) sequences. Experimental validation and in-depth genomic analysis and annotations, however, are required to validate the presence or absence of these and other ORFs on the spike protein vaccine candidate sequences.

## Additional Steps to Exclude Overlapping ORFs on DNA/RNA Vaccine Sequences

Alternatively to codon optimization, another option for safeguarding against overlapping ORFs in a vaccine candidate is to select short section(s) of the protein sequence that code for the most antigenic regions, as exemplified by the Pfizer BNT162b1 mRNA vaccine that codes for a trimeric construct of the receptor-binding domain of the SARS-CoV-2 spike protein (74). A shorter sequence may have a lower potential to code for other smaller proteins. ORF predictions using the NCBI ORFfinder on the nucleotide sequences corresponding to the receptor-binding domain (nucleotides 999-1569 on wild-type spike sequence) of the wild-type, BNT162b2, and mRNA-1273 spike proteins revealed the presence of three (29-36 aa), two (28 and 44 aa), and zero ORFs on alternative frames, respectively, and three (29-69 aa), six (27-53 aa), and one (170 aa) ORFs, respectively, when considering alternative initiation codons. Although the shortening of the spike sequence reduces the number of overlapping ORFs, the potential for alternative translation still remains.

Multimeric vaccine DNA/RNA sequences that include antigenic regions of different viral proteins could also be used to increase immunogenicity while shortening the length of the construct and, thus, controlling for the presence of overlapping ORFs (75–77). For example, the hepatitis C E2 protein scaffold has been used to present the antigenic HIV-1 gp120 variable loop region to promote immunogenicity for potential HIV vaccination (78). Thus, the downsizing of the sequence to include only the most antigenic regions of the spike receptor-binding domain, such as the receptor-binding motif, or domains from other viral proteins to be placed on a codon-optimized protein scaffold may further control for overlapping protein-coding sequences (79). Sequence length and content can further affect the number of overlapping ORFs, but the scrutinization of protein-coding regions nevertheless relies on validating the translation of alternative reading frames.

The use of experimental techniques, such as ribosomal profiling or mass spectrometry, on vaccinated patient or laboratory animal samples or pseudovirus-infected tissue cultures may help determine whether the overlapping ORFs are translated and to what degree they are translated compared to the intended protein. Thus, several potential checkpoints can be utilized to control for the translation of small ORFs within DNA/RNA vaccine candidate sequences. Otherwise, unintended proteins could be translated by the host cell, which may lead to side effects resembling that of viral infection symptoms.

## Conclusions

DNA/RNA vaccines have proven to be an effective way to develop vaccines quickly for emerging pathogens. However, with a new set of solutions, comes a new set of problems (80). Although the wild-type SARS-CoV-2 spike protein nucleotide sequence has been found to code for translated overlapping genes, ORF detection predictions on the sequences of two mRNA vaccines reveal that codon optimization has the potential to disrupt non-specific translation. Additional overlapping ORFs can arise during codon optimization; thus, the final sequences should nevertheless be scrutinized for their protein-coding potential. In the case of DNA vaccines and viral vectors, the negative-sense strand should also be checked for its protein-coding potential. Additionally, as variants of concern become known and vaccines are altered to include them, the spontaneous generation of ORFs should be re-assessed. Many precautionary steps have been taken to ensure the safety and efficacy of the mRNA vaccines, including nucleoside modification to reduce inflammatory responses and 5’-capping and polyadenylation tail length optimization to increase mRNA stability and translation (20). Thus, the inclusion of additional steps to ensure that vaccine sequences code solely for the intended protein may also lead to better health and safety outcomes. Measures to check for other adverse effects on host cells, such as those resulting from potential interactions of vaccine nucleotide sequences with host RNAs or proteins, or the host microbiome may be increase efficacy and safety as well (81)⁠. More in-depth investigation of these delivery methods may reveal aspects that should be further refined to safeguard against unintended side effects.

## Data Availability Statement

The original contributions presented in the study are included in the article/supplementary material. Further inquiries can be directed to the corresponding author.

## Author Contributions

CB, MB, and AV contributed to conception and design of the study. CB, MB, and AV contributed to sequence and structural analyses. All authors contributed to manuscript writing and revision. All authors contributed to the article and approved the submitted version.

## Funding

TB thanks the Wellcome Trust for support through an Investigator Award (200814/Z/16/Z; 2016 -2021). CB was supported by Antibiotic Research UK (PHZJ/687).

## Conflict of Interest

The authors declare that the research was conducted in the absence of any commercial or financial relationships that could be construed as a potential conflict of interest.

## Publisher’s Note

All claims expressed in this article are solely those of the authors and do not necessarily represent those of their affiliated organizations, or those of the publisher, the editors and the reviewers. Any product that may be evaluated in this article, or claim that may be made by its manufacturer, is not guaranteed or endorsed by the publisher.

## References

[1] Wu F Zhao S Yu B Chen Y-M Wang W Song Z-G. A New Coronavirus Associated With Human Respiratory Disease in China. Nature (2020) 579:265–9. doi: 10.1038/s41586-020-2008-3 PMC7094943 32015508

[2] Cevik M Kuppalli K Kindrachuk J Peiris M. Virology, Transmission, and Pathogenesis of SARS-CoV-2. BMJ (2020) 371:m3862. doi: 10.1136/bmj.m3862 33097561

[3] Cucinotta D Vanelli M. WHO Declares COVID-19 a Pandemic. Acta BioMed (2020) 91:157–60. doi: 10.23750/abm.v91i1.9397 PMC7569573 32191675

[4] Gorain B Choudhury H Molugulu N Athawale RB Kesharwani P. Fighting Strategies Against the Novel Coronavirus Pandemic: Impact on Global Economy. Front Public Heal (2020) 8:606129. doi: 10.3389/fpubh.2020.606129 PMC7756013 33363098

[5] Pronker ES Weenen TC Commandeur H Claassen EHJHM Osterhaus ADME. Risk in Vaccine Research and Development Quantified. PloS One (2013) 8:e57755. doi: 10.1371/journal.pone.0057755 23526951 PMC3603987

[6] Scannell JW Blanckley A Boldon H Warrington B. Diagnosing the Decline in Pharmaceutical R&D Efficiency. Nat Rev Drug Discov (2012) 11:191–200. doi: 10.1038/nrd3681 22378269

[7] Brisse M Vrba SM Kirk N Liang Y Ly H. Emerging Concepts and Technologies in Vaccine Development. Front Immunol (2020) 11:583077. doi: 10.3389/fimmu.2020.583077 33101309 PMC7554600

[8] Nelson CW Ardern Z Goldberg TL Meng C Kuo C-H Ludwig C. Dynamically Evolving Novel Overlapping Gene as a Factor in the SARS-CoV-2 Pandemic. Elife (2020) 9:e59633. doi: 10.7554/eLife.59633 33001029 PMC7655111

[9] Finkel Y Mizrahi O Nachshon A Weingarten-Gabbay S Morgenstern D Yahalom-Ronen Y. The Coding Capacity of SARS-CoV-2. Nature (2021) 589:125–30. doi: 10.1038/s41586-020-2739-1 32906143

[10] Davidson AD Williamson MK Lewis S Shoemark D Carroll MW Heesom KJ. Characterisation of the Transcriptome and Proteome of SARS-CoV-2 Reveals a Cell Passage Induced in-Frame Deletion of the Furin-Like Cleavage Site From the Spike Glycoprotein. Genome Med (2020) 12:68. doi: 10.1186/s13073-020-00763-0 32723359 PMC7386171

[11] Lan J Ge J Yu J Shan S Zhou H Fan S. Structure of the SARS-CoV-2 Spike Receptor-Binding Domain Bound to the ACE2 Receptor. Nature (2020) 581:215–20. doi: 10.1038/s41586-020-2180-5 32225176

[12] Dandan L Jinming L Suzanne KC. Immunologic Testing for SARS-CoV-2 Infection From the Antigen Perspective. J Clin Microbiol (2021) 59:e02160-20. doi: 10.1128/JCM.02160-20 33318065 PMC8091849

[13] Grant OC Montgomery D Ito K Woods RJ. Analysis of the SARS-CoV-2 Spike Protein Glycan Shield Reveals Implications for Immune Recognition. Sci Rep (2020) 10:14991. doi: 10.1038/s41598-020-71748-7 32929138 PMC7490396

[14] Watanabe Y Allen JD Wrapp D McLellan JS Crispin M. Site-Specific Glycan Analysis of the SARS-CoV-2 Spike. Science (2020) 369:330–3. doi: 10.1126/science.abb9983 PMC7199903 32366695

[15] Samrat SK Tharappel AM Li Z Li H. Prospect of SARS-CoV-2 Spike Protein: Potential Role in Vaccine and Therapeutic Development. Virus Res (2020) 288:198141. doi: 10.1016/j.virusres.2020.198141 32846196 PMC7443330

[16] Wang J Peng Y Xu H Cui Z. Williams RO 3rd. The COVID-19 Vaccine Race: Challenges and Opportunities in Vaccine Formulation. AAPS PharmSciTech (2020) 21:225. doi: 10.1208/s12249-020-01744-7 32761294 PMC7405756

[17] Peletta A Prompetchara E Tharakhet K Kaewpang P Buranapraditkun S Techawiwattanaboon T. DNA Vaccine Administered by Cationic Lipoplexes or by *In Vivo* Electroporation Induces Comparable Antibody Responses Against SARS-CoV-2 in Mice. Vaccines (2021) 9:874. doi: 10.3390/vaccines9080874 34451998 PMC8402479

[18] Rijkers GT Weterings N Obregon-Henao A Lepolder M Dutt TS van Overveld FJ. Antigen Presentation of mRNA-Based and Virus-Vectored SARS-CoV-2 Vaccines. Vaccines (2021) 9:848. doi: 10.3390/vaccines9080848 34451973 PMC8402319

[19] Heinz FX Stiasny K. Distinguishing Features of Current COVID-19 Vaccines: Knowns and Unknowns of Antigen Presentation and Modes of Action. NPJ Vaccines (2021) 6:104. doi: 10.1038/s41541-021-00369-6 34400651 PMC8368295

[20] Park JW Lagniton PNP Liu Y Xu R-H. mRNA Vaccines for COVID-19: What, Why and How. Int J Biol Sci (2021) 17:1446–60. doi: 10.7150/ijbs.59233 PMC8071766 33907508

[21] Chung H He S Nasreen S Sundaram ME Buchan SA Wilson SE. Effectiveness of BNT162b2 and mRNA-1273 Covid-19 Vaccines Against Symptomatic SARS-CoV-2 Infection and Severe Covid-19 Outcomes in Ontario, Canada: Test Negative Design Study. BMJ (2021) 374:n1943. doi: 10.1136/bmj.n1943 34417165 PMC8377789

[22] Pritchard E Matthews PC Stoesser N Eyre DW Gethings O Vihta K-D. Impact of Vaccination on New SARS-CoV-2 Infections in the United Kingdom. Nat Med (2021) 27:1370–8. doi: 10.1038/s41591-021-01410-w PMC8363500 34108716

[23] Wibmer CK Ayres F Hermanus T. SARS-CoV-2 501Y.V2 Escapes Neutralization By South African COVID-19 Donor Plasma. Nat Med (2021) 27:622–5. 10.1038/s41591-021-01285-x 33654292

[24] Hosseini SA Zahedipour F Mirzaei H Kazemi Oskuee R. Potential SARS-CoV-2 Vaccines: Concept, Progress, and Challenges. Int Immunopharmacol (2021) 97:107622. doi: 10.1016/j.intimp.2021.107622 33895475 PMC8006194

[25] FDA Approves First COVID-19 Vaccine (2021). Available at: https://www.fda.gov/news-events/press-announcements/fda-approves-first-covid-19-vaccine (Accessed September 17, 2021).

[26] Chakraborty S Mallajosyula V Tato CM Tan GS Wang TT. SARS-CoV-2 Vaccines in Advanced Clinical Trials: Where Do We Stand? Adv Drug Deliv Rev (2021) 172:314–38. doi: 10.1016/j.addr.2021.01.014 PMC7816567 33482248

[27] Menni C Klaser K May A Polidori L Capdevila J Louca P. Vaccine Side-Effects and SARS-CoV-2 Infection After Vaccination in Users of the COVID Symptom Study App in the UK: A Prospective Observational Study. Lancet Infect Dis (2021) 21:939–49. doi: 10.1016/S1473-3099(21)00224-3 PMC8078878 33930320

[28] Lv G Yuan J Xiong X Li M. Mortality Rate and Characteristics of Deaths Following COVID-19 Vaccination. Front Med (2021) 8:670370. doi: 10.3389/fmed.2021.670370 PMC8160119 34055843

[29] Karayeva E Kim HW Bandy U Clyne A Marak TP. Monitoring Vaccine Adverse Event Reporting System (VAERS) Reports Related to COVID-19 Vaccination Efforts in Rhode Island. R I Med J (2013) (2021) 104:64–6. 34437669

[30] Deb A Abdelmalek J Iwuji K Nugent K. Acute Myocardial Injury Following COVID-19 Vaccination: A Case Report and Review of Current Evidence From Vaccine Adverse Events Reporting System Database. J Prim Care Commun Health (2021) 12:21501327211029230. doi: 10.1177/21501327211029230 PMC8255555 34219532

[31] Sharifian-Dorche M Bahmanyar M Sharifian-Dorche A Mohammadi P Nomovi M Mowla A. Vaccine-Induced Immune Thrombotic Thrombocytopenia and Cerebral Venous Sinus Thrombosis Post COVID-19 Vaccination; a Systematic Review. J Neurol Sci (2021) 428:117607. doi: 10.1016/j.jns.2021.117607 34365148 PMC8330139

[32] Martinez-Marmol R Giordano-Santini R Kaulich E Cho A-N Riyadh MA Robinson E. The SARS-CoV-2 Spike (S) and the Orthoreovirus P15 Cause Neuronal and Glial Fusion. bioRxiv (2021), 2021.09.01.458544. doi: 10.1101/2021.09.01.458544

[33] Buzhdygan TP DeOre BJ Baldwin-Leclair A Bullock TA McGary HM Khan JA. The SARS-CoV-2 Spike Protein Alters Barrier Function in 2D Static and 3D Microfluidic *In-Vitro* Models of the Human Blood–Brain Barrier. Neurobiol Dis (2020) 146:105131. doi: 10.1016/j.nbd.2020.105131 33053430 PMC7547916

[34] Olajide OA Iwuanyanwu VU Adegbola OD Al-Hindawi AA. SARS-CoV-2 Spike Glycoprotein S1 Induces Neuroinflammation in BV-2 Microglia. bioRxiv (2021), 2020.12.29.424619. doi: 10.1101/2020.12.29.424619 PMC8551352 34709564

[35] Keith M Tapas P Vijayamahantesh Ranjit R Tom G. SARS-CoV-2 Spike Protein Induces Paracrine Senescence and Leukocyte Adhesion in Endothelial Cells. J Virol (2021) 95:e00794-21. doi: 10.1128/JVI.00794-21 PMC8354225 34160250

[36] Orr MW Mao Y Storz G Qian S-B. Alternative ORFs and Small ORFs: Shedding Light on the Dark Proteome. Nucleic Acids Res (2020) 48:1029–42. doi: 10.1093/nar/gkz734 PMC7026640 31504789

[37] Calviello L Hirsekorn A Ohler U. Quantification of Translation Uncovers the Functions of the Alternative Transcriptome. Nat Struct Mol Biol (2020) 27:717–25. doi: 10.1038/s41594-020-0450-4 32601440

[38] Huang J-Z Chen M Chen D Gao X-C Zhu S Huang H. A Peptide Encoded by a Putative lncRNA HOXB-AS3 Suppresses Colon Cancer Growth. Mol Cell (2017) 68:171–84.e6. doi: 10.1016/j.molcel.2017.09.015 28985503

[39] Anderson DM Anderson KM Chang C-L Makarewich CA Nelson BR McAnally JR. A Micropeptide Encoded by a Putative Long Noncoding RNA Regulates Muscle Performance. Cell (2015) 160:595–606. doi: 10.1016/j.cell.2015.01.009 25640239 PMC4356254

[40] Dinman JD. Mechanisms and Implications of Programmed Translational Frameshifting. WIREs RNA (2012) 3:661–73. doi: 10.1002/wrna.1126 PMC3419312 22715123

[41] Bazykin GA Kochetov AV. Alternative Translation Start Sites Are Conserved in Eukaryotic Genomes. Nucleic Acids Res (2011) 39:567–77. doi: 10.1093/nar/gkq806 PMC3025576 20864444

[42] Yang Y Wang Z. IRES-Mediated Cap-Independent Translation, a Path Leading to Hidden Proteome. J Mol Cell Biol (2019) 11:911–9. doi: 10.1093/jmcb/mjz091 PMC6884710 31504667

[43] Chirico N Vianelli A Belshaw R. Why Genes Overlap in Viruses. Proc Biol Sci (2010) 277:3809–17. doi: 10.1098/rspb.2010.1052 PMC2992710 20610432

[44] Pavesi A. Origin, Evolution and Stability of Overlapping Genes in Viruses: A Systematic Review. Genes (Basel) (2021) 12:809. doi: 10.3390/genes12060809 34073395 PMC8227390

[45] Michel CJ Mayer C Poch O Thompson JD. Characterization of Accessory Genes in Coronavirus Genomes. Virol J (2020) 17:131. doi: 10.1186/s12985-020-01402-1 32854725 PMC7450977

[46] Weingarten-Gabbay S Klaeger S Sarkizova S Pearlman LR Chen DY Gallagher KM. Profiling SARS-CoV-2 HLA-I Peptidome Reveals T Cell Epitopes From Out-of- Frame ORFs. Cell (2021) 184(15):3962–80. 10.1016/j.cell.2021.05.046 PMC8173604 34171305

[47] Pavesi A. Prediction of Two Novel Overlapping ORFs in the Genome of SARS-CoV-2. Virology (2021) 562:149–57. doi: 10.1016/j.virol.2021.07.011 PMC8317007 34339929

[48] Jungreis I Sealfon R Kellis M. SARS-CoV-2 Gene Content and COVID-19 Mutation Impact by Comparing 44 Sarbecovirus Genomes. Nat Commun (2021) 12:2642. doi: 10.1038/s41467-021-22905-7 33976134 PMC8113528

[49] Aoki A Adachi H Mori Y Ito M Sato K Okuda K. A Rapid Screening Assay for L452R and T478K Spike Mutations in SARS-CoV-2 Delta Variant Using High-Resolution Melting Analysis. J Toxicol Sci (2021) 46:471–6. doi: 10.2131/jts.46.471 34602531

[50] Gordon DE Jang GM Bouhaddou M Xu J Obernier K White KM. A SARS-CoV-2 Protein Interaction Map Reveals Targets for Drug Repurposing. Nature (2020) 583:459–68. doi: 10.1038/s41586-020-2286-9 PMC7431030 32353859

[51] Dominguez Andres A Feng Y Campos AR Yin J Yang C-C James B. SARS-CoV-2 ORF9c Is a Membrane-Associated Protein That Suppresses Antiviral Responses in Cells. bioRxiv (2020), 2020.08.18.256776. doi: 10.1101/2020.08.18.256776

[52] Alsulami AF Thomas SE Jamasb AR Beaudoin CA Moghul I Bannerman B. SARS-CoV-2 3D Database: Understanding the Coronavirus Proteome and Evaluating Possible Drug Targets. Brief Bioinform (2021) 22:769–80. doi: 10.1093/bib/bbaa404 PMC7929435 33416848

[53] Beaudoin CA Jamasb AR Alsulami AF Copoiu L van Tonder AJ Hala S. Predicted Structural Mimicry of Spike Receptor-Binding Motifs From Highly Pathogenic Human Coronaviruses. Comput Struct Biotechnol J (2021) 19:3938–53. doi: 10.1016/j.csbj.2021.06.041 PMC8249111 34234921

[54] Šali A Blundell TL. Comparative Protein Modelling by Satisfaction of Spatial Restraints. J Mol Biol (1993) 234:779–815. doi: 10.1006/jmbi.1993.1626 8254673

[55] Wang S Li W Liu S Xu J. RaptorX-Property: A Web Server for Protein Structure Property Prediction. Nucleic Acids Res (2016) 44:W430–5. doi: 10.1093/nar/gkw306 PMC4987890 27112573

[56] Wang S Ma J Xu J. AUCpreD: Proteome-Level Protein Disorder Prediction by AUC-Maximized Deep Convolutional Neural Fields. Bioinformatics (2016) 32:i672–9. doi: 10.1093/bioinformatics/btw446 PMC5013916 27587688

[57] Yang J Anishchenko I Park H Peng Z Ovchinnikov S Baker D. Improved Protein Structure Prediction Using Predicted Interresidue Orientations. Proc Natl Acad Sci (2020) 117:1496–503. doi: 10.1073/pnas.1914677117 PMC6983395 31896580

[58] Bartas M Volná A Beaudoin CA Poulsen ET Červeň J Brázda V. Unheeded SARS-CoV-2 Proteins? A Deep Look Into Negative-Sense RNA. bioRxiv (2021), 2020.11.27.400788. doi: 10.1101/2020.11.27.400788 PMC9116216 35229157

[59] Gabler F Nam S-Z Till S Mirdita M Steinegger M Söding J. Protein Sequence Analysis Using the MPI Bioinformatics Toolkit. Curr Protoc Bioinforma (2020) 72:e108. doi: 10.1002/cpbi.108 33315308

[60] Lu S Wang J Chitsaz F Derbyshire MK Geer RC Gonzales NR. CDD/SPARCLE: The Conserved Domain Database in 2020. Nucleic Acids Res (2020) 48:D265–8. doi: 10.1093/nar/gkz991 PMC6943070 31777944

[61] Krogh A Larsson B von Heijne G Sonnhammer EL. Predicting Transmembrane Protein Topology With a Hidden Markov Model: Application to Complete Genomes. J Mol Biol (2001) 305:567–80. doi: 10.1006/jmbi.2000.4315 11152613

[62] Ayoub R Lee Y. Rupee: A Fast and Accurate Purely Geometric Protein Structure Search. PloS One (2019) 14:1–17. doi: 10.1371/journal.pone.0213712 PMC6420038 30875409

[63] McClenaghan C Hanson A Lee S-J Nichols CG. Coronavirus Proteins as Ion Channels: Current and Potential Research. Front Immunol (2020) 11:573339. doi: 10.3389/fimmu.2020.573339 33154751 PMC7586316

[64] Cagliani R Forni D Clerici M Sironi M. Coding Potential and Sequence Conservation of SARS-CoV-2 and Related Animal Viruses. Infect Genet Evol (2020) 83:104353. doi: 10.1016/j.meegid.2020.104353 32387562 PMC7199688

[65] Peisach E Pabo CO. Constraints for Zinc Finger Linker Design as Inferred From X-Ray Crystal Structure of Tandem Zif268–DNA Complexes. J Mol Biol (2003) 330:1–7. doi: 10.1016/S0022-2836(03)00572-2 12818197

[66] Mauro VP Chappell SA. A Critical Analysis of Codon Optimization in Human Therapeutics. Trends Mol Med (2014) 20:604–13. doi: 10.1016/j.molmed.2014.09.003 PMC4253638 25263172

[67] Madeira F Park YM Lee J Buso N Gur T Madhusoodanan N. The EMBL-EBI Search and Sequence Analysis Tools APIs in 2019. Nucleic Acids Res (2019) 47:W636–41. doi: 10.1093/nar/gkz268 PMC6602479 30976793

[68] Bourret J Alizon S Bravo IG. COUSIN (COdon Usage Similarity INdex): A Normalized Measure of Codon Usage Preferences. Genome Biol Evol (2019) 11:3523–8. doi: 10.1093/gbe/evz262 PMC6934141 31800035

[69] Puigbò P Bravo IG Garcia-Vallve S. CAIcal: A Combined Set of Tools to Assess Codon Usage Adaptation. Biol Direct (2008) 3:38. doi: 10.1186/1745-6150-3-38 18796141 PMC2553769

[70] Sharp PM Li WH. The Codon Adaptation Index–a Measure of Directional Synonymous Codon Usage Bias, and its Potential Applications. Nucleic Acids Res (1987) 15:1281–95. doi: 10.1093/nar/15.3.1281 PMC340524 3547335

[71] Li Y Yang X Wang N Wang H Yin B Yang X. GC Usage of SARS-CoV-2 Genes Might Adapt to the Environment of Human Lung Expressed Genes. Mol Genet Genomics (2020) 295:1537–46. doi: 10.1007/s00438-020-01719-0 PMC7473593 32888056

[72] Dilucca M Forcelloni S Georgakilas AG Giansanti A Pavlopoulou A. Codon Usage and Phenotypic Divergences of SARS-CoV-2 Genes. Viruses (2020) 12:498. doi: 10.3390/v12050498 PMC7290700 32366025

[73] Smith TRF Patel A Ramos S Elwood D Zhu X Yan J. Immunogenicity of a DNA Vaccine Candidate for COVID-19. Nat Commun (2020) 11:2601. doi: 10.1038/s41467-020-16505-0 32433465 PMC7239918

[74] Walsh EE Frenck RW Jr. Falsey AR Kitchin N Absalon J Gurtman A. Safety and Immunogenicity of Two RNA-Based Covid-19 Vaccine Candidates. N Engl J Med (2020) 383:2439–50. doi: 10.1056/NEJMoa2027906 PMC7583697 33053279

[75] Aebischer A Wernike K König P Franzke K Wichgers Schreur PJ Kortekaas J. Development of a Modular Vaccine Platform for Multimeric Antigen Display Using an Orthobunyavirus Model. Vaccines (2021) 9:651. doi: 10.3390/vaccines9060651 34203630 PMC8232151

[76] Denis J Majeau N Acosta-Ramirez E Savard C Bedard M-C Simard S. Immunogenicity of Papaya Mosaic Virus-Like Particles Fused to a Hepatitis C Virus Epitope: Evidence for the Critical Function of Multimerization. Virology (2007) 363:59–68. doi: 10.1016/j.virol.2007.01.011 17320136

[77] Sahin U Oehm P Derhovanessian E Jabulowsky RA Vormehr M Gold M. An RNA Vaccine Drives Immunity in Checkpoint-Inhibitor-Treated Melanoma. Nature (2020) 585:107–12. doi: 10.1038/s41586-020-2537-9 32728218

[78] Trovato M Maurano F D’Apice L Costa V Sartorius R Cuccaro F. E2 Multimeric Scaffold for Vaccine Formulation: Immune Response by Intranasal Delivery and Transcriptome Profile of E2-Pulsed Dendritic Cells. BMC Microbiol (2016) 16:152. doi: 10.1186/s12866-016-0772-x 27421762 PMC4947308

[79] Yi C Sun X Ye J Ding L Liu M Yang Z. Key Residues of the Receptor Binding Motif in the Spike Protein of SARS-CoV-2 That Interact With ACE2 and Neutralizing Antibodies. Cell Mol Immunol (2020) 17:621–30. doi: 10.1038/s41423-020-0458-z PMC7227451 32415260

[80] Forni G Mantovani A Forni G Mantovani A Moretta L Rappuoli R. COVID-19 Vaccines: Where We Stand and Challenges Ahead. Cell Death Differ (2021) 28:626–39. doi: 10.1038/s41418-020-00720-9 PMC7818063 33479399

[81] Gonçalves E Guillén Y Lama JR Sanchez J Brander C Paredes R. Host Transcriptome and Microbiota Signatures Prior to Immunization Profile Vaccine Humoral Responsiveness. Front Immunol (2021) 12:657162. doi: 10.3389/fimmu.2021.657162 34040607 PMC8141841

